# Alcohol consumption and alcohol counselling behaviour among US medical students: cohort study

**DOI:** 10.1136/bmj.a2155

**Published:** 2008-11-07

**Authors:** Erica Frank, Lisa Elon, Timothy Naimi, Robert Brewer

**Affiliations:** 1 University of British Columbia, School of Population and Public Health, and Department of Family Practice, 5804 Fairview Avenue, Vancouver, BC, Canada; 2 Emory University School of Medicine, Department of Family and Preventive Medicine, 49 Jesse Hill Jr Drive, Atlanta, GA 30303, USA; 3 Emory University Rollins School of Public Health, Department of Biostatistics and Bioinformatics, 1518 Clifton Road, Atlanta, GA 30322; 4 National Center for Chronic Disease Prevention and Health Promotion, Centers for Disease Control and Prevention, Mail stop k-67, 4770 Buford Highway NE, Atlanta, GA 30341

## Abstract

**Objective** To determine which factors affect alcohol counselling practices among medical students.

**Design** Cohort study.

**Setting** Nationally representative medical schools (n=16) in the United States.

**Participants** Medical students who graduated in 2003.

**Interventions** Questionnaires were completed (response rate 83%) at the start of students’ first year (n=1846/2080), entrance to wards (typically during the third year of training) (n=1630/1982), and their final (fourth) year (n=1469/1901).

**Main outcome measures** Previously validated questions on alcohol consumption and counselling.

**Results** 78% (3777/4847) of medical students reported drinking in the past month, and a third (1668/ 4847) drank excessively; these proportions changed little over time. The proportion of those who believed alcohol counselling was highly relevant to care of patients was higher at entrance to wards (61%; 919/1516) than in final year students (46%; 606/1329). Although students intending to enter primary care were more likely to believe alcohol counselling was highly relevant, only 28% of final year students (391/1393) reported usually or always talking to their general medical patients about their alcohol consumption. Excessive drinkers were somewhat less likely than others to counsel patients or to think it relevant to do so. In multivariate models, extensive training in alcohol counselling doubled the frequency of reporting that alcohol counselling would be clinically relevant (odds ratio 2.3, 95% confidence interval 1.6 to 3.3) and of reporting doing counselling (2.2, 1.5 to 3.3).

**Conclusions** Excessive drinking and binge drinking among US medical students is common, though somewhat less prevalent than among comparably aged adults in the US general population. Few students usually discussed alcohol use with patients, but greater training and confidence about alcohol counselling predicted both practising and believing in the relevance of alcohol counselling. Medical schools should consider routinely training students to screen and counsel patients for alcohol misuse and consider discouraging excessive drinking.

## Introduction

Each year, excessive alcohol consumption kills about 79 000 people in the United States,^1^ making it the third leading preventable cause of death.^2^ Clinical alcohol screening and brief counselling help to reduce excessive consumption and related harms and are therefore recommended by the US Preventive Services Task Force.^3^ Such counselling is among the most effective and cost effective clinical preventive services.^4^ In the US, however, few health providers ask patients about their alcohol use,^5^ ^6^ despite about 75% of excessive drinkers having current health insurance and reporting having a check up within the past 24 months.^7^ While several factors contribute to low counselling rates, many physicians are unaware of guidelines for low risk drinking and harmful levels of alcohol consumption, and many feel ill prepared to counsel their patients.^8^ It is not clear how medical students’ experiences and drinking behaviours might relate to their opinions or subsequent practices.

Drinking behaviours among medical students have important implications for the health of the general population. Firstly, physicians and future physicians are important opinion leaders and role models in terms of health related behaviours. Secondly, medical students’ own drinking behaviours might shape their beliefs about levels of consumption that are normal or safe, particularly in the absence of specific knowledge about evidence based drinking guidelines. Finally, the drinking behaviours of medical students might influence their attitudes and comfort about counselling those who drink excessively; there is a strong and consistent relation between physicians’ personal health practices and their counselling practices,^9^ including their practices around alcohol.^10^ Understanding any association between medical students’ alcohol counselling habits and their drinking patterns and educational experiences could help increase screening and brief counselling interventions among healthcare providers.

We investigated the drinking habits of medical students and any association between these habits and personal, professional, and school based characteristics. We also examined whether a belief that alcohol counselling was highly relevant to intended specialty and self reported frequency of alcohol counselling were associated with variables that could be influenced in medical school.

## Design

### Study design

All medical students graduating in 2003 at 16 US medical schools were eligible to complete three questionnaires during their medical training: at first year orientation (summer/autumn 1999), entrance to wards (typically in their third year), and in their final year. 

A convenience sample of 17 US medical schools participated in the study; one school was excluded for non-adherence to the protocol. The 16 remaining schools were relatively representative of all US medical schools in terms of student age (first year student average age 24 *v* 24 nationally), school size (students per school 563 *v* 527 nationally), medical school research ranking from the US National Institutes of Health (school average 64 *v* 62 nationally), private/public school balance (51% private schools *v* 41% nationally), under-represented minorities (13% black, Hispanic, and Native American *v* 11% nationally), sex (45% women *v* 43% nationally), and geographical distribution.^11^ ^12^ ^13^ ^14^

Students’ responses were linked across time with a unique identifier consisting of mother’s initials at her birth and father’s first two initials. At entry into medical school, 2080 students were eligible to complete the survey and 1846 responded (89%); 1982 were eligible at entry to wards (that is, during either their second or third year) and 1630 responded (82%); 1901 were eligible at the final year and 1469 responded (77%). Of the 2316 students who provided responses, 72% (n=1658) did so at more than one time point; 971 responded at three time points, 687 at two, and 658 at one. Time specific school response rates ranged from 48% to 98%, with 83% responding overall. Not all students were eligible and able to respond at all three survey points (for example, because of pursuing a complementary degree).

Questionnaires were usually administered after semi-mandatory activities (such as after exams, during orientation lunches, or at the end of a class) to encourage participation; students were informed that questionnaires were anonymous and confidential, and participation was voluntary. At some schools with lower response rates, we used Dillman’s five stage mailing process^15^ to increase rates; surveys completed with this enhanced follow-up accounted for 5% of the final year responses. School participation was encouraged by offering school specific data (in aggregate and without student identifiers) to school investigators. The median item non-response rate was 3%, with lower rates for demographic information (<1%), 2% missing on drinking variables, and between 5-10% on counselling variables. All available information was used in each analysis.

### Description of variables

The three questions on alcohol have been used in a large national US survey and have been validated previously.^16^ The questions were:

- During the past month, on about how many days did you drink any alcoholic beverage?
- On the days when you drank, about how many drinks did you drink, on average? (a drink is one can or bottle of beer or wine cooler [chilled wine with juice or water], one glass of wine, one cocktail, or one shot of liquor)
- How often in the past month did you have five or more drinks on one occasion?

Based on responses to these questions, medical students were classified into one of three groups: excessive drinkers, non-excessive drinkers, or non-drinkers. Consumption was classified as “excessive” in the previous month if it met at least one of these criteria: reported at least one occasion on which they consumed five or more drinks (that is, reported one or more episodes of binge drinking), or men who drank more than two drinks a day on average or women who drank more than one drink a day on average, based on their responses to the frequency and average quantity questions. This criterion corresponds to the at risk average drinking levels specified by the US National Institute for Alcohol Abuse and Alcoholism.^17^ Drinkers who did not meet criteria for excessive drinkers were defined as non-excessive drinkers, and those who drank no alcohol in the past month were classified as non-drinkers. We calculated the prevalence of excessive drinking by dividing the number of excessive drinkers by the total number of students, and multiplying by 100.

Smoking questions were drawn from the same source.^16^

- Have you smoked at least 100 cigarettes in your entire life?
- Do you NOW smoke cigarettes every day, some days, or not at all?
- How long has it been since you quit smoking cigarettes?
- During the past 30 days, on the days you smoked, how many cigarettes did you smoke per day?

We also asked how many days of the past 30 did they

- Smoke part or all of a cigar
- Use any chewing tobacco, dip, or snuff
- Smoke any tobacco in a pipe
- Smoke part or all of a cigarette.

Our primary professional outcomes were two variables concerning medical students’ counselling of patients on alcohol: the perceived relevance of alcohol counselling in the student’s intended practice (“How relevant do you think talking to patients about alcohol will be in your intended practice?”) and validated^18^ frequency of alcohol counselling (“With a typical general medicine patient, how often do you actually talk to patients about alcohol?”). The possible response categories for relevance were “not at all,” “somewhat,” and “highly.” For frequency of counselling the response categories were “never-rarely,” “sometimes,” and “usually-always.” We asked about relevance of counselling at all time points, while frequency was measured only in the final year questionnaire, as these students are actually involved in clinical care.

We assessed drinking and counselling behaviours based on characteristics that are known to affect drinking and counselling behaviours, including demographics, personal health related habits, opinions on prevention, intended specialty, and school environment relating to personal health promotion. We also asked (in two separate questions) about apparent attitudes of their medical school and peers towards medical students’ alcohol use.

### Statistical analysis

We tested the bivariate associations between our three outcomes (drinking, counselling relevance, and counselling frequency) and independent variables with χ^2^ test. Because of the number of associations being tested, we limited our discussion of significant results to those with P<0.01. We calculated sex adjusted odds ratios for excessive drinking and crude odds ratios for relevance and frequency of alcohol counselling, with their 95% confidence intervals, with generalised estimating equations with robust variance estimation.

We used logistic regression analysis to assess the relation between the perceived relevance and frequency of counselling with three independent variables: the amount of training in alcohol counselling (“extensive” versus less than extensive); the degree of agreement with the statement, “Physicians have a responsibility to promote prevention with their patients”; and alcohol consumption in the past month. We adjusted for potential confounders, including sex and current intended specialty. For the analysis, perceived relevance of alcohol related counselling was dichotomised as highly relevant versus any lesser response and the frequency of counselling as usually-always counselling versus less often or no counselling. All measurements were reported on the senior year questionnaire. We used SUDAAN software,^19^ which is designed for the analysis of clustered data, for all data analysis, treating each school as a cluster and each student’s multiple responses as subclusters in the analyses. Model parameters were estimated with a SUDAAN procedure by using working exchangeable generalised estimating equations with robust variance estimation. Model fit was also assessed via standardised deviance residuals and the Hosmer-Lemeshow goodness of fit test.^20^

## Results

Most (78%, 3777/4847) students reported drinking alcohol in the past month, and 34% (1668/4847) drank excessively (540/2227 (24%) women and 1126/2615 (43%) men); these proportions changed little over time at medical school (table 1). Of those who met the criteria for excessive drinkers, nearly all (99%, 1661/1668) reported binge drinking at least once in the past month, and 36% (594/1668) reported three or more binge episodes (data not shown). In the subset who responded at all time points, 86% (267/310) of those reporting excessive drinking at year one made the same report on at least one subsequent questionnaire; 59% (184/310) reported heavy drinking at all points. Four fifths of first year non-drinkers (167/208) reported no drinking on at least one other questionnaire, and only 13 later reported excessive drinking.

**Table 1 tbl1:** Alcohol consumption* among US medical students (1999-2003) and its association with demographic characteristics

Characteristic	No of students	Alcohol consumption in past month			χ^2^ P value†	Adjusted odds ratio (95% CI)‡
		None (%)	Non-excessive (%)	Excessive (%)		
Overall	4847§	22	44	34		
Time point:						
First year	1818	22	44	33	0.5	1.0
Introduction to wards (third year)	1601	23	43	34		1.0 (0.9 to 1.1)
Final year	1428	21	43	37		1.2 (0.97 to 1.4)
Intended specialty:						
Primary care	1669	26	47	27	0.001	1.0
Not primary care	2444	20	41	39		1.4 (1.2 to 1.6)
Undecided	690	22	43	36		1.3 (0.98 to 1.8)
Sex:						
Female	2227	23	53	24	0.0001	1.0
Male	2615	21	36	43		2.4 (2.0 to 2.8)
Ethnicity:						
Black/African American	376	42	47	11	<0.0001	1.0
Asian	906	32	43	25		2.2 (1.2 to 4.2)
Hispanic	204	15	52	32		3.2 (1.2 to 8.4)
White	3107	17	43	40		4.5 (2.4 to 8.7)
Other	239	26	40	34		3.4 (1.5 to 7.6)
Marital status:						
Married	924	33	46	22	0.0001	1.0
Unmarried couple	273	11	54	35		2.5 (1.6 to 4.0)
Single/widowed/divorced	3618	20	42	38		2.5 (2.0 to 3.1)
Tobacco use in past month:						
None, never smoked	3538	28	48	24	<0.0001	1.0
Past smoker	286	13	45	42		2.4 (1.6 to 3.5)
Light/infrequent	835	2	30	68		6.2 (4.9 to 7.9)
>10 cigarettes/day or >19 days with any tobacco	175	4	21	75		8.9 (5.3 to 14.8)
Strength of religious identity:						
Very strong	932	46	40	14	0.0001	1.0
Strong	1202	24	45	31		2.9(2.3 to 3.7)
Moderate	1293	15	44	41		4.6 (3.3 to 6.5)
Low	845	8	43	48		6.1 (4.2 to 8.9)
None	546	15	47	38		4.0 (2.5 to 6.3)
Stress in past two weeks:						
A lot	1054	25	45	31	0.06	1.0
Moderate	1927	21	46	32		1.2 (0.96 to 1.4)
Little/none	1831	21	40	39		1.4 (1.02 to 1.9)
Stress in past 12 months:						
A lot	1568	23	45	33	0.004	1.0
Moderate	2316	22	45	33		1.2 (1.1 to 1.5)
Little/none	922	20	38	41		1.7 (1.2 to 2.3)

*Consumption was classified as “excessive” in previous month if it met at least one of these criteria: reported at least one occasion on which ≥5 drinks consumed (that is, reported one or more episodes of binge drinking), or >2 drinks/day on average in men or >1 drink/day on average in women, based on responses to frequency and average quantity questions. Those who drank less than excessively were classified as non-excessive drinkers. Those reporting no alcohol consumption in past month were classified as non-drinkers.

†χ^2^ test for association in contingency tables.

‡Odds ratio of excessive drinking (*v* non-excessive/non-drinkers) compared with reference group (listed with odds ratio=1.0), controlled for sex.

§Sum of observations in various strata might not sum to total number of observations (4945) because of non-response.

Although similar proportions of students who were intending to practise in primary and non-primary care drank alcohol, non-primary care students were more likely to drink excessively; this was true even after we adjusted for sex disparity in specialty choice (table 1).[Table tbl1] And although almost equal proportions of men and women drank alcohol, men were more than twice as likely to drink excessively. By race, black or African-American students were least likely to drink alcohol and drink excessively, while white students were most likely to do so. When stratified by relationship status, married students were least likely to drink or to drink excessively.

Several personal habits and beliefs were associated with drinking habits (table 1).[Table tbl1] Nearly all current smokers reported drinking alcohol, and they were nearly three times more likely to drink excessively than those who had never smoked (69% (699/1010) *v* 24% (841/3538)). Strong religious identity was associated with more abstention and less excessive use of alcohol. Rates of excessive drinking were highest among those reporting lower stress levels.

Those who did not drink excessively agreed more strongly that one would provide better counselling if one abstained or did not drink excessively. Academic and peer environment were also associated with drinking behaviours (table 2); there was a modest relation between students’ perception of school attitude regarding alcohol and the report of excessive drinking. The odds of excessive drinking were 60% higher if peers’ attitude was that “drinking is a good release” compared with “no obvious attitude.” There was a strong relation between a school’s support for non-drinking and students’ drinking behaviours; however, this relation was strongly influenced by findings from one religiously affiliated school that discouraged alcohol use. There were no significant relations (data not shown, P>0.7) between attitudes of school or peers versus perceived relevance or frequency of alcohol counselling.

**Table 2 tbl2:** Alcohol consumption among US medical students (1999-2003) and its association with attitudinal and environmental characteristics

Characteristic	No of students	Alcohol consumption in past month			χ^2^ P value*	Adjusted odds ratio (95% CI)†
		None (%)	Non-excessive (%)	Excessive (%)		
“I will be able to provide more credible and effective counselling if I drink alcohol in moderation or not at all”:						
Strongly agree	313	27	48	25	0.0001	1.0
Agree	744	21	45	34		1.5 (1.1 to 2.1)
Neither agree/disagree	218	17	38	45		2.3 (1.5 to 3.3)
Disagree/strongly disagree	83	8	27	65		5.0 (2.9 to 8.7)
Peers’ attitudes toward alcohol use‡:						
No obvious attitude	314	26	43	31	0.03	1.0
We shouldn’t drink	57	46	25	30		0.9 (0.2 to 3.6)
We should drink in moderation	1225	23	46	31		1.1 (0.7 to 1.7)
Drinking is a good release	1316	19	41	39		1.6 (1.3 to 2.0)
School’s attitude toward alcohol‡:						
No obvious attitude	832	20	47	34	0.006	1.0
We shouldn’t drink	356	55	29	15		0.3 (0.1 to 0.8)
Drink in moderation	1426	15	47	39		1.3 (1.03 to 1.6)
Drinking is a good release	305	24	35	42		1.3 (0.96 to 1.7)

*χ^2^ test for association in contingency tables.

†Odds ratio of excessive drinking (*v* non-excessive/non-drinkers) compared with reference group (odds ratio=1.0), controlled for sex.

‡Questions asked only at introduction to wards and during final year.

Among excessive drinkers, about three quarters of men and women drank on 14 or fewer days in the past month (table 3). On the days excessive drinkers consumed alcohol, 62% (696/1124) of men drank three or more drinks and 89% (478/539) of women drank two or more drinks. Furthermore, 18% (201/1124) of male and 22% (117/539) of female excessive drinkers reported that their usual alcohol consumption on days they drank met or exceeded binge levels (that is, five or more drinks for men, four or more drinks for women). Among excessive drinkers, 61% (691/1126) of men and 44% (229/520) of women reported binge drinking on multiple occasions in the past 30 days.

**Table 3 tbl3:** Drinking characteristics of US medical students (1999-2003) who consume alcohol, by sex and category of alcohol consumption*

Drinking characteristic (past month)	Non-excessive (%) (n=2108)			Excessive (%) (n=1666)	
	Men (n=937)	Women (n=1171)		Men (n=1126)	Women (n=540)
Days of drinking:					
1-4	63	64		17	17
5-9	24	24		35	38
10-14	9	9		24	22
15-19	2	2		10	9
20-24	2	0		8	7
≥25	1	0		5	6
Usual No of drinks per drinking day:					
1	50	55		9	11
2	37	37		29	42
3	11	8		28	25
4	3	0		16	15
≥5	N/A	N/A		18	7
No of occasions with ≥5 drinks:					
1	N/A	N/A		39	56
2-3	N/A	N/A		33	30
4-5	N/A	N/A		15	9
≥6	N/A	N/A		13	5

N/A=not applicable.

*Consumption classified as “excessive” in previous month if it met at least one of: reported at least one occasion on which ≥5 drinks consumed (that is, reported one or more episodes of binge drinking), or drank >2 drinks/day on average in men or >1 drink/day on average in women, based on responses to frequency and average quantity questions. Those who drank less than excessively were classified as non-excessive drinkers. Those reporting no alcohol consumption in past month were classified as non-drinkers.

When asked how relevant speaking to patients about alcohol would be in their intended medical practice, students intending a primary care specialty were significantly more likely to answer “highly relevant”; this was the case for all time points in school (P<0.01, table 4). During their years of clinical training, students’ perception that alcohol counselling was highly relevant declined from 76% (330/432) to 59% (241/406) among those intending to pursue primary care specialties, and from 52% (397/767) to 39% (344/886) among those intending non-primary care specialties. The proportion of students reporting high confidence in alcohol counselling increased from 42% (638/1507) at orientation to wards to 50% (686/1363) during the final year. Only a minority at either entrance to wards (19%; 294/1510) or the final year (35%; 458/1321) thought they had extensive training in alcohol counselling, although nearly all said they had at least some training by senior year. Overall, 28% (391/1393) of seniors reported usually or always talking to their general medical patients about their alcohol consumption.

**Table 4 tbl4:** Alcohol counselling by US medical students (1999-2003): self reports on relevance to intended practice, training, confidence, and frequency. Figures are numbers (percentages) of students

Counselling question	First year	Orientation to wards	Final year	χ^2^ P (for time)
**How relevant do you think talking to patients about alcohol will be in your intended practice?**				
Total (all specialties):				
Not at all	106 (6)	56 (4)	150 (12)	<0.002
Somewhat	734 (40)	531 (35)	563 (42)	
Highly	970 (54)	919 (61)	606 (46)	
Primary care:				
Not at all	36 (5)	4 (1)	5 (1)	<0.0001*
Somewhat	279 (35)	98 (23)	160 (39)	
Highly	476 (60)	330 (76)	241 (59)	
Non-primary care:				
Not at all	56 (9)	45 (6)	154 (17)	0.003*
Somewhat	288 (44)	325 (42)	388 (44)	
Highly	315 (48)	397 (52)	344 (39)	
**How confident are you about talking to patients about alcohol?†**				
Not at all	—	63 (4)	20 (1)	0.001
Somewhat	—	806 (53)	657 (48)	
Highly	—	638 (42)	686 (50)	
**How much training have you had on talking to patients about alcohol?†**				
None	—	129 (9)	26 (2)	0.0003
Some	—	1087 (72)	837 (63)	
Extensive	—	294 (19)	458 (35)	
**With a typical general medicine patient, how often do you actually perform this activity?‡**				
Never/rarely	—	—	110 (8)	—
Sometimes	—	—	892 (64)	
Usually/always	—	—	391 (28)	

*In addition to significant χ^2^, proportion responding “highly” (*v* “less than highly”) followed significant quadratic trend over time.

†Queried during orientation to wards and during final year.

‡Queried only during final year.

Nearly half (46%; 606/1330) of final year students considered alcohol counselling highly relevant to their intended specialty (tables 5 and 6) [Table tbl6]. Characteristics associated with reporting higher perceived relevance of alcohol counselling included: being female; being of black/African American race; abstaining from alcohol in the past month; intending to go into primary care; having extensive training in the topic; feeling highly confident in alcohol counselling; strongly agreeing that moderation in their personal drinking habits was associated with credible and effective alcohol counselling; strongly agreeing that counselling on healthy lifestyles is effective; expressing more interest in prevention than treatment; and agreeing that physicians have a responsibility to promote prevention.

**Table 5 tbl5:** Relation of personal characteristics with perceived relevance to intended practice and self reported frequency of counselling general medicine patients, reported during senior year of US medical school (2003)

	Highly relevant to practice				Usually/always counsel		
	No of students*	% of students	Crude OR (95% CI)		No of students*	% of students	Crude OR (95% CI)
Total	1330	46 (41 to 51)			1394	28 (24 to 31)	
Sex:							
Men	704	39	1.0 (ref)		739	26	1.0 (ref)
Women	625	53	1.8 (1.4 to 2.2)		653	31	1.3 (0.97 to 1.6)
Ethnicity:							
White	847	42	1.0 (ref)		887	25	1.0 (ref)
Asian	253	49	1.4 (1.0 to 1.8)		257	34	1.6 (1.1 to 2.2)
Black/African American	109	62	2.3 (1.4 to 3.9)		116	39	1.9 (1.1 to 3.5)
Hispanic	55	47	1.2 (0.6 to 2.5)		57	30	1.3 (0.9 to 1.8)
Other	64	47	1.2 (0.8 to 1.9)		73	30	1.3 (0.9 to 1.9)
Alcohol drinking in past month†:							
None	278	55	1.7 (1.1 to 2.7)		291	32	1.4 (1.03 to 2.0)
Non-excessive	567	45	1.2 (0.9 to 1.5)		589	30	1.3 (0.96 to 1.8)
Excessive	465	42	1.0 (ref)		494	24	1.0 (ref)

*Denominator for percentage displayed. Numbers for various characteristics might not add up to row total because of item non-response for those characteristics.

†Consumption classified as “excessive” in previous month if it met at least one of: reported at least one occasion on which ≥5 drinks consumed (that is, reported one or more episodes of binge drinking), or if men drank >2 drinks/day on average or if women drank >1 drink/day on average, based on responses to frequency and average quantity questions. Those who drank less than excessively were classified as non-excessive drinkers. Those reporting no alcohol consumption in past month were classified as non-drinkers.

**Table 6 tbl6:** Relation of professional characteristics with perceived relevance (n=1330) to intended practice and self reported frequency of counselling general medicine patients (n=1394) reported during final year of US medical school (2003)

	Highly relevant to practice				Usually/always counsel		
	No of students*	% of students	Crude OR (95% CI)		No of students*	% of students	Crude OR (95% CI)
Intended specialty:							
Primary care	406	59	2.3 (1.8 to 3.0)		421	29	1.1 (0.8 to 1.4)
Non-primary care	887	39	1.0 (ref)		935	27	1.0 (ref)
Training in alcohol counselling:							
Extensive	451	59	2.3 (1.7 to 3.2)		455	40	2.3 (1.6 to 3.3)
Less than extensive	835	39	1.0 (ref)		857	22	1.0 (ref)
Confidence in alcohol counselling:							
Highly confident	663	55	2.2 (1.6 to 3.0)		680	36	2.5 (1.7 to 3.6)
Less than highly	631	36	1.0 (ref)		672	19	1.0 (ref)
I will be able to provide more credible and effective counselling if I drink alcohol in moderation or not at all:							
Strongly agree	306	57	1.0 (ref)		317	37	1.0 (ref)
Agree	721	44	0.6 (0.4 to 0.8)		747	26	0.6 (0.5 to 0.8)
Neither	197	33	0.4 (0.2 to 0.6)		214	21	0.4 (0.3 to 0.7)
Disagree/strongly disagree	79	48	0.7 (0.5 to 1.1)		81	301	0.7 (0.4 to 1.4)
Patients are more likely to adopt healthier lifestyles if physicians counsel them to do so:							
Strongly agree	232	55	1.0 (ref)		239	38	1.0 (ref)
Agree	810	44	0.7 (0.5 to 0.9)		844	25	0.6 (0.4 to 0.8)
Neither	176	39	0.5 (0.3 to 0.9)		190	28	0.7 (0.3 to 1.3)
Disagree/ strongly disagree	79	48	0.8 (0.4 to 1.4)		80	28	0.6 (0.3 to 1.3)
I am less interested in prevention than treatment:							
Strongly agree	77	32	1.0 (ref)		82	28	1.0 (ref)
Agree	293	39	1.3 (0.8 to 2.2)		308	26	0.9 (0.6 to 1.4)
Neither	285	39	1.3 (0.8 to 2.2)		302	25	0.9 (0.5 to 1.5)
Disagree	488	48	2.0 (1.02 to 3.7)		503	27	0.9 (0.5 to 1.7)
Strongly disagree	156	70	4.8 (2.2 to 10.5)		159	41	1.8 (0.95 to 3.3)
Physicians have a responsibility to promote prevention with their patients:							
Strongly agree	340	56	2.3 (1.5 to 3.5)		352	36	2.6 (1.3 to 5.1)
Agree	831	43	1.4 (1.05 to 1.8)		858	26	1.6 (0.8 to 3.0)
Neither disagree/ strongly disagree	124	35	1.0 (ref)		142	18	1.0 (ref)

*Denominator for percent displayed. Numbers for various characteristics might not sum to n for relevance or frequency because of item non-response for those characteristics.

Characteristics associated with frequent alcohol counselling among senior medical students included: being Asian or black/African American; abstaining from alcohol or drinking non-excessively; having extensive training in the topic; feeling highly confident in alcohol counselling; strongly agreeing that moderation in their personal drinking habits is associated with credible and effective alcohol counselling; strongly agreeing that counselling on healthy lifestyles is effective; and strongly agreeing that physicians have a responsibility to promote prevention. Sex, intended specialty, and interest in prevention compared with treatment were not significantly associated with frequency of counselling. Among those who disagreed that moderation in their personal drinking habits was associated with credible and effective alcohol counselling, 19% (15/81) rarely or never counselled, compared with 7% (92/1277) of others (data not shown).

In multivariate models, extensive training in alcohol counselling was associated with over twice the odds of a final year student’s belief that alcohol counselling would be relevant to their practice (adjusted odds ratio 2.3, 95% confidence interval 1.6 to 3.3) and reporting that they often counselled patients about their alcohol consumption (2.2, 1.5 to 3.3) (table 7). Personal alcohol consumption (that is, being either a non-drinker or non-excessive drinker) and belief in one’s responsibility to promote prevention were somewhat (but non-significantly) associated with talking with patients about alcohol consumption. Intention to go into a primary care specialty and being female were associated with higher odds of reporting high relevance of alcohol counselling but were not significantly associated with higher odds of talking to patients about alcohol.

**Table 7 tbl7:** Multivariate testing of association of training, drinking, and prevention attitudes with perceived relevance and self reported frequency of alcohol counselling among US medical school seniors (2002-3). Odds ratios are adjusted* and presented with 95% confidence intervals and P values†

	Relevance to intended specialty (n=1215)	Frequency of counselling (n=1237)
**Modifiable characteristics**		
Training in alcohol counselling:		
None/some	1.0	1.0
Extensive	2.3 (1.6 to 3.3)	2.2 (1.5 to 3.3)
P value	0.0002	0.0006
Alcohol drinking in past month:		
Excessive	1.0	1.0
Non-excessive	1.0 (0.7 to 1.3)	1.2 (0.8 to 1.9)
None	1.4 (0.9 to 2.3)	1.4 (0.9 to 2.1)
P value	0.1	0.3
Physicians have a responsibility to promote prevention with their patients:		
Strongly agree	1.4 (0.8 to 2.4)	1.9 (0.8 to 4.4)
Agree	1.1 (0.7 to 1.6)	1.3 (0.6 to 2.7)
Neutral/disagree/strongly disagree	1.0	1.0
P value	0.09	0.08
**Control variables**		
Sex:		
Female	1.5 (1.2 to 1.9)	1.1 (0.8 to 1.4)
Male		1.0
P value	0.002	0.6
Current intended specialty:		
Primary care	2.2 (1.8 to 2.8)	1.0 (0.7 to 1.3)
Non-primary care	1.0	1.0
P value	<0.0001	0.9

*Odds of reporting “highly relevant” or “usually/always” compared with reference group, adjusted for all other variables listed.

†Satterthwaite adjusted F test for significance of covariate adjusted relation of characteristic with outcome.

## Discussion

### Summary

In this study of alcohol consumption among US medical students about a third (24% of women and 43% of men) reported excessive drinking in the previous month, typically in the form of binge drinking, and usually (among excessive drinkers) on multiple occasions, with frequencies changing little over time at medical school. Only a quarter of final year students routinely assessed their patients’ drinking behaviours, and students’ perceptions of the relevance and frequency of performing alcohol counselling were significantly associated with their training in alcohol counselling; only half of final year students reported that they were highly confident about their ability to discuss alcohol with their patients.

### Comparison of medical students’ alcohol consumption with other populations and previous literature

Our findings are consistent with earlier studies of drinking among US medical students.^21^ ^22^ ^23^ ^24^ For example, a study in the mid-1980s of 341 first year medical students at four schools found that 44% were either “frequent” or “heavy” drinkers; these categories involved either occasional or frequent consumption that included binge drinking.^25^ This bingeing prevalence was about 50% higher than that reported in a survey of 548 medical students from eight US medical schools in the mid-1990s.^26^ Studies in the United Kingdom,^27^ Germany,^28^ and New Zealand^29^ showed rates of bingeing in medical students similar to or higher than rates in the US.

Our reported rates of binge drinking were lower than the 51% of 18-24 year olds, and 40% of 25-34 year old Americans in 2002 who reported consuming five or more drinks at least once in the past month^7^ and lower than the rates in 1999 US college seniors (45% in the past two weeks, both for highly competitive colleges and for all final year students).^30^ They were, however, substantially higher than bingeing rates reported by female doctors aged 30-70 (0.1% in the past month)^10^ and did not decline during medical school.

Overall, 79% of male and 77% of female medical students reported consuming alcohol in the past 30 days. Consistent with other data showing lower rates of abstention with higher socioeconomic status, these rates of use were slightly higher than national rates: in the US 77% of men and 65% of women aged 25-44 consumed alcohol in 2001.^31^ The only previous national data collected on alcohol use among medical students (conducted among 2046 final year students in 1987) found that 87% of women and 88% of men reported alcohol use in the previous month, with no assessments of frequency or quantity.^21^ Similar numbers (83% of women and 88% of men) were reported in 1987 for residents’ alcohol use in the past month, also without assessments of frequency or quantity.^32^

While bingeing rates in medical students were lower than their peers’, bingeing is still a prevalent behaviour. This suggests that, as with their poor dietary habits (but in contrast to their relatively good smoking and physical activity habits),^33^ alcohol use has not yet been sufficiently addressed as an important health risk behaviour among US medical students.

### Correlates of medical students’ alcohol counselling practices

The relatively low rate of US medical students who provide alcohol counselling is also of concern. Screening and counselling for alcohol misuse in adults in primary care, including non-dependent excessive drinking (such as binge drinking), are strongly recommended by the US Preventive Services Task Force^3^ and are consistent with the conclusions of the Institute of Medicine about the importance of “broadening the base” of alcohol counselling to include all patterns of alcohol consumption that increase the risk of health problems.^34^ Furthermore, screening and brief counselling interventions are particularly high impact and cost effective clinical preventive services,^4^ and a high proportion of conditions leading to medical visits and inpatient admissions are alcohol related or are exacerbated by excessive drinking.^35^ ^36^ Despite the efficiency of counselling, our finding that less than a third of students routinely counsel general medical patients about their alcohol consumption is consistent with studies that show only a small proportion of patients are screened by physicians about their alcohol use.^5^ ^37^ Our findings are also consistent with other studies pointing to a lack of adequate or appropriate training in alcohol counselling as an important contributor to low rates of screening.^8^ ^38^ ^39^ ^40^ More encouragingly, like some other behaviours studied in this population and elsewhere,^41^ we found that more training was an important predictor of subsequent preventive counselling behaviour among senior medical students.

In bivariate analysis, excessive drinking was associated with lower perceived relevance and frequency of alcohol counselling; findings were similar but not significant in multivariate analysis. A relation between personal and clinical practices has been found among US physicians for many behaviours, including drinking alcohol and counselling patients about alcohol: only 32% of female physicians who reported drinking more than two drinks a week (the 75th centile for alcohol intake) typically counselled patients about alcohol at least once a year compared with 42% of those who drank two or fewer drinks a week (P<0.001 for the difference between the two groups’ counselling rates).^10^ Similarly, bivariate analyses showed that students whose peers who did not encourage drinking as a release, and those attending a school that encouraged abstention, were somewhat less likely to drink excessively; this behaviour could be considered part of a professionalism curriculum.^42^

### Strengths and weaknesses

Our study’s strengths include a high response rate, a large and representative sample, the longitudinal design, and linked data over time. Our findings, however, are also subject to some limitations. Although not all students responded during all three time periods, response rates for each time period exceeded 75%. Data are limited by being survey data (rather than qualitative or interview) from schools with variations within and between them and from self report. Our question on frequency of alcohol counselling, however, was validated with extensive standardised testing,^18^ with a strong relation shown between medical students’ self reported alcohol counselling and their actual counselling, as reported by standardised patients. While we have validated some of our data on personal health practice,^43^ rates of binge drinking are difficult to validate and are likely to be underestimated because of bias due to social desirability, particularly among health professionals. In addition, we did not use a sex specific definition of binge drinking and might have underestimated the prevalence of binge drinking among women.

Medical students’ personal and clinical attitudes about alcohol have important implications for their current care of patients. Furthermore, drinking practices in young adulthood help to establish patterns for later drinking.^44^ As medical school environments might influence students’ consumption (through both the formal and informal or hidden curriculum),^45^ it might be useful to consider whether efforts should be made to alter the drinking environments at US medical schools to discourage excessive drinking (though this might be difficult to accomplish).^46^ ^47^ If medical students are better educated about guidelines for low risk drinking and screening and counselling for alcohol misuse, they might be more likely to adhere to clinical prevention guidelines and be better equipped to identify and reduce excessive drinking among their patients. Medical schools should also consider supporting the implementation of effective interventions to reduce excessive drinking among medical students and the general population.^48^ ^49^ ^50^

**What is already known on this topic**

Screening and brief counselling help to reduce excessive alcohol consumption and related harms
Drinking behaviours among medical students have important implications for the health of the general population

**What this study adds**

The prevalence of binge drinking among US medical students is somewhat lower than their peers in the general population, though still quite high and substantially higher than rates reported by US female physicians
Few medical students usually discussed alcohol with patients, but as personal and educational characteristics are associated with their perceived relevance and frequency of counselling, interventions on these characteristics could improve students’ rates of alcohol counselling

## Notes

Contributors: EF was responsible for the conception, design, and interpretation of data, for drafting the article and revising it, and providing final approval of the version to be published. She is guarantor. LE contributed to study design, was responsible for data analysis and interpretation, contributed to article revision for important intellectual content, and approved the final version. TN contributed to study design, analysis and interpretation of data, drafting components of the article, critical revisions, and approved the final version. RB contributed to study design, analysis and interpretation of data, drafting components of the article, critical revisions, and approved the final version.

Ethical approval: This work was approved by the institutional review board at Emory University, and informed consent was given by all participants.

Provenance and peer review: Not commissioned; externally peer reviewed.

## Notes

**Cite this as: ***BMJ* 2008;337:a2155
